# Supplementary material for: Hepatic transcriptome profiling unveils candidate genes in cattle with liver abscesses under the influence of beef genetics in dairy cattle
Source: PLoS One. 2025 Jun 9;20(6):e0324209. doi: 10.1371/journal.pone.0324209 (PMC12148182; doi:10.1371/journal.pone.0324209)
Supplement: Attachment — Submitted filename: Response to Reviewers Felizari PlosOne.docx [file pone.0324209.s008.docx]

299 Here and throughout the manuscript and tables the authors use the work abscesses. However, the methods indicate that the hepatic tissue taken, was from the same region on the right side of the lobe. Were the samples from abscessed livers taken in the area of an abscess OR was the tissue sampled without regard for abscess presence in the tissue. In other words, did the sample “select” for abscessed tissue or did the tissue sample look normal? Regardless, “staining in abscessed livers, “ would read better.

We thank the reviewer’s comment and apologize for the confusion. To clarify, we collected the same region from every animal without regard for the abscess presence in the tissue that was evaluated by scoring the whole liver. All the samples collected didn’t present any visible abscess, as the presence of an abscess could have introduced bacterial contamination, potentially affecting sequencing results. To make the text more comprehensive, we added a small statement in the method section to clarify that at line 106.

We also changed line 300, we changed it to: “staining in abscessed livers.”

327 This may be a journal style issue but, “Kumar et al. [39]

We agree this style doesn’t help the reader, so we changed it to: “A study……. [39]”.

395 … responses is interpreted to suggest?

We corrected the sentence now at line 396 with “…this may suggest…”

396 … conducted by Hulllahalli et al. …[52]

Also, here we updated the text, of this citation with: “In a study conducted in mice…[52]”.

412 This comparison needs to be clarified. Higher than what?

We thank the reviewer for the suggestion. We then changed the sentence in line 413 to make it more understandable for the reader as follows:” In addition, our study showed higher circulating levels of IGF-I in beef × dairy steers compared to the levels in straightbred beef cattle found in other studies [9, 10]. IGF-I levels in our animals were found to be…”

435 Perturbations ?

We corrected with perturbations.

Figures Can the quality of these images be improved. And Abscesses vs. Abscessed Livers

We thank the reviewer for the suggestion. We provided an updated version of the same representative images where a higher contrast of the Dapi and the IGF-IR staining will help to visualize better the differences between the groups. However, Figures 2, 3, and 4 made on R, it is not possible to increase their quality since we already saved them in higher quality as TIF File.

We apologize, and we agree that this can be confusing for the readers. We then modified the Figures (1 and 5) by changing the group abscesses to “Abscessed Livers” and healthy to “Healthy Livers”. We also changed the figure legends and the text accordingly.
